# Low-Carbon Monoxide Diffusing Capacity, Patient-Reported Measures and Reduced Nailfold Capillary Density Are Associated with Interstitial Lung Disease in Systemic Sclerosis

**DOI:** 10.3390/jpm14060635

**Published:** 2024-06-14

**Authors:** Rossella De Angelis, Edoardo Cipolletta, Francesca Francioso, Marina Carotti, Sonia Farah, Andrea Giovagnoni, Fausto Salaffi

**Affiliations:** 1 Rheumatology Unit, Department of Clinical and Molecular Sciences, Polytechnic University of Marche, “Carlo Urbani” Hospital, Jesi, 60035 Ancona, Italy; edoardo.cipolletta@nottingham.ac.uk (E.C.); frafrancioso@gmail.com (F.F.); sonia.farah91@gmail.com (S.F.); fausto.salaffi@gmail.com (F.S.); 2 IRCCS INRCA, 60121 Ancona, Italy; 3 Academic Rheumatology, University of Nottingham, Nottingham NG7 2RD, UK; 4 Department of Radiology, “Ospedali Riuniti”, Polytechnic University of Marche, 60121 Ancona, Italy; m.carotti@staff.univpm.it (M.C.); a.giovagnoni@univpm.it (A.G.)

**Keywords:** systemic sclerosis, interstitial lung disease, risk factors

## Abstract

The aim of this paper is to identify factors associated with interstitial lung disease (ILD) in patients with systemic sclerosis (SSc) and build an algorithm to better define this association for a personalised application in clinical practice. Methods. A total of 78 SSc patients underwent HRCT to assess ILD. Demographic, clinical and laboratory variables were collected, focusing on those associated either directly or indirectly with lung involvement. The discriminant value of each variable was determined using the operating characteristic curves (ROC) and included in a model to estimate the strength of ILD association in SSc. Results. Thirty-three (42.31%) patients showed ILD on HRCT. DLco, M-Borg, GERD-Q and capillary density were significantly associated with the presence of ILD-SSc. A model including these variables had a coefficient of determination (R^2^) of 0.697. DLco had an AUC of 0.861 (*p* < 0.001) with a cut-off of ≤72.3% (sensitivity 78.8%, specificity 91.1%, +LR 8.86). The m-Borg Scale showed an AUC of 0.883 (*p* < 0.001) with a cut-off >2 (sensitivity 84.8%, specificity 82.2%, +LR 4.77), GERD-Q had an AUC of 0.815 (*p* < 0.001) with a cut-off >7 (sensitivity 72.7%, specificity 86.7%, +LR 5.45). The capillary density showed an AUC of 0.815 (*p* < 0.001) with a cut-off of ≤4.78 (sensitivity 87.9%, specificity 68.9%, +LR 2.82). Based on the pre-test probability values, these four variables were applied to Fagan’s nomogram to calculate the post-test probability of this association. Conclusions. Our study identified four associated clinical factors of ILD in SSc patients. Moreover, their inclusion in an algorithm for the post-test probability, tailored to the specific patients’ characteristics, significantly increases the ability to find out the presence of SSc-ILD.

## 1. Introduction

Systemic sclerosis (SSc) is a chronic connective tissue disorder characterised by widespread fibrosis and impaired microcirculation [1,2]. Endothelial and fibroblast dysfunction leading to tissue hypoxia, together with altered immune responses, play key roles in disease pathogenesis [1,2]. In SSc, fibrosis and vasculopathy are closely associated and result in heterogeneous multi-organ clinical manifestations with a variable prognosis [1,2].

In addition to the skin, internal organs are often affected, particularly the lungs [2], representing one of the major causes of death in SSc [3,4]. SSc interstitial lung disease (SSc-ILD) is characterised by early pulmonary infiltration of inflammatory cells, mainly B cells, promoting the generation and persistence of myofibroblasts, accumulation of excess extracellular matrix and subsequent fibrosis of the lung parenchyma [2,5].

All patients with SSc are at risk of SSc-ILD. Established risk factors for SSc-ILD are respiratory symptoms, smoking history, ethnicity (Native American, African heritage), male gender, diffuse cutaneous (dcSSc) subset and anti-topo I/Scl-70 antibodies [3,6]. Although the probability seems to be higher in the early stages of the disease [3,7], a recent study regarding 826 patients with SSc-ILD from the EUSTAR database found that only 21% of patients had a disease duration of < 3 years, and about half of the SSc patients had no evident risk factors for SSc-ILD [8]. Therefore, the identification of additional factors associated with SSc-ILD remains an open research question [3,7,8].

The primary tool for diagnosing SSc-ILD is high-resolution computed tomography-HRCT [6,9]. Moreover, baseline and longitudinal changes in pulmonary function tests (PFTs), mostly forced vital capacity (FVC) and diffusing capacity of the lungs for carbon monoxide (DLco) [4,6,9], are found to be associated with SSc-ILD. Other measures directly related to ILD in SSc have been recently investigated, i.e., patient-reported measures of symptoms severity and quality of life [3,10,11], and the extent of oesophageal involvement [11,12]. Furthermore, the usefulness of nailfold videocapillaroscopy (NVC) in SSc-ILD has been addressed [13,14], highlighting that abnormal NVC patterns (i.e., active and late) are linked with the presence of SSc-ILD, and emphasising that NVC could potentially be used as a biomarker in screening algorithms for SSc-ILD [13,14]. Nevertheless, none of the available tools alone can detect SSc-ILD with appropriate sensitivity and specificity compared to HRCT [3,6,7,9].

Therefore, we aimed to identify additional factors mainly linked to the clinical/biometric interface of pulmonary tissue damage that might be associated with the presence of ILD in SSc patients, trying to build a useful algorithm for further strengthening and personalising this association in daily clinical practice.

## 2. Patients and Methods

The study population consisted of 78 non-consecutive outpatients with SSc, without a known diagnosis of ILD, aged ≥ 18 years, defined by the current classification criteria [15], enrolled between January and November 2023. Written informed consent was obtained from all participants, in accordance with the Declaration of Helsinki.

Patients who were unable to undergo NVC (due to reduced visibility or amputation of the phalanges), or unable to perform PFTs, patients with a high probability of PAH according to the DETECT algorithm [16], and/or with PAH diagnosed by right heart catheterisation RHC, patients with SSc overlapping with other systemic connective tissue diseases, and/or those with chronic lung diseases other than SSc-ILD and those taking antifibrotic drugs were excluded from the study.

Study approval was obtained in the context of a cross-sectional database of patients with RP secondary to SSc (Raynaud Phenomenon Database, RAYMOND study, n. 257-2020 ID 1650). According to the study intentions, and in agreement with the current recommendations of SSc-ILD [3,6], all patients had periodic HRCT scans to screen ILD, typically every 12–24 months, depending on their symptoms and the clinical phenotype. 

### 2.1. Demographic, Clinical and Laboratory Data

Basic information included age, sex and disease duration (from the first non-Raynaud symptom). Patients were classified into limited (lcSSc) and diffuse SSc (dcSSc) [17]. None fit the strict definition of SSc ‘sine scleroderma’ [17]. The modified Rodnan skin score (mRSS) was used to assess the extent of skin thickness. The score was calculated by summing the rating from all 17 areas (range 0–51) [18]. We gathered other clinical data such as oesophageal symptoms (dysphagia, reflux) and dyspnoea, antinuclear antibodies (ANA), anti-extractable nuclear antigens (anti-ENA), SSc-related (anti-centromere/CENP-B, anti-topoisomerase I/Scl70, anti-RNA polymerase III) and non-SSc related (anti-U3 RNP, anti-SSA) antibodies.

### 2.2. HRCT Assessment and Visual Reader-Based Disease Quantification

All HRCT examinations were performed according to a standard protocol, using a CT 64GE Light-speed VCT power scanner with a rotation tube scanning time of 0.65 s. Scans were acquired in full inspiration from the apex to the lung base in the supine position, at 120 kV and 300 mAs, and slice thickness and spacing of scans of 1.25 and 7 mm, respectively. Contrast media agents were not employed.

The HRCTs were evaluated independently by a radiologist (MC—consultant with 20 years of experience in the field of musculoskeletal radiology) and a rheumatologist (FS—trained in HRCT interpretation) blinded to clinical and respiratory functional findings. In case of disagreement, a third reader (AG—expert thoracic radiologist) examined the scans to obtain a final consensus decision. The intraclass correlation coefficient (ICC) of the radiologists’ agreement level on total HRCT scores, as previously calculated by the CoVR method, was 0.80 [19,20]. The lung parenchymal abnormalities were assessed according to the Warrick score [21]. A point value was assigned to each abnormality as follows: ground-glass appearance = 1, irregular pleural margins = 2, septal/subpleural lines = 3, honeycombing = 4 and subpleural cysts = 5. In each patient, the “severity of disease” score was obtained by adding single point values. An “extent of disease” score was obtained by counting the number of bronchopulmonary segments involved for each abnormality: one to three segments scored as 1; four to nine segments scored as 2; and more than nine segments scored as 3. The severity and extent of the disease were then calculated as the total HRCT score (range from 0 to 30). To estimate intra-observer reliability, each reader examined all HRCTs twice, with an interval of at least four weeks.

### 2.3. Patient-Reported Measures

The following patient-centred measures were obtained: dyspnoea severity using the modified Borg Dyspnoea Scale (m-Borg) by the interviewer-administered paper version [20,22], a numerical scale for assessing perceived dyspnoea (breathing discomfort) with a scale of 0 = no breathlessness at all, 0.5 = very, very slight (just noticeable), 1 = very slight, 2 = slight breathlessness, 3 = moderate, 4 = somewhat severe, 5 = severe breathlessness, 7 = very severe breathlessness, 9 = very, very severe (almost maximum) and 10 = maximum, and the Health Assessment Questionnaire-Disability Index (HAQ-DI) [20,23], which is a generic, self-administrated patient-reported outcome (PRO) instrument targeting people with musculoskeletal impairment, defined as a condition-specific measure of functional status (assessing activities of daily living) [20,23]. The standard HAQ-DI is calculated as an ordinal variable, from 0 = no disability to 3 = severe disability. 

Gerd-Q is a simple self-administered questionnaire consisting of six items for assessing the risk of gastroesophageal reflux disease—GERD [24]. Four of these assess symptoms and situations considered to be positive predictors for GERD—heartburn, regurgitation, sleep disturbances (caused by gastric symptoms) and taking antacids, in addition to those medically prescribed—while the remaining two assess symptoms that are considered to be negative predictors for reflux (nausea and epigastric discomfort). The answers refer to the frequency of symptoms over the past week, using a Likert scale of 0 to 3 for positive characteristics, and 3 to 0 for negative characteristics [25]. The maximum score that can be achieved is 18. A GERD-Q cut-off of 9 was associated with the best ratio of sensitivity and specificity for the diagnosis of gastro-esophageal reflux [25].

### 2.4. Pulmonary Function Tests

PFTs were performed within 2 weeks from the HRCT scan by a flow-sensing spirometer and a body plethysmograph connected to a computer for data analysis while the patient was at rest in a seated position. These tests consisted of spirometry using a computerised lung analyser (MasterScreen Diffusion, Jaeger GmbH, Höchber, Germany). FVC (% predicted value) and DLco (% predicted value, corrected for haemoglobin) were obtained. At least three measurements were taken for each variable to guarantee repeatability [20].

### 2.5. Nailfold Capillaroscopy 

**Figure 1:**
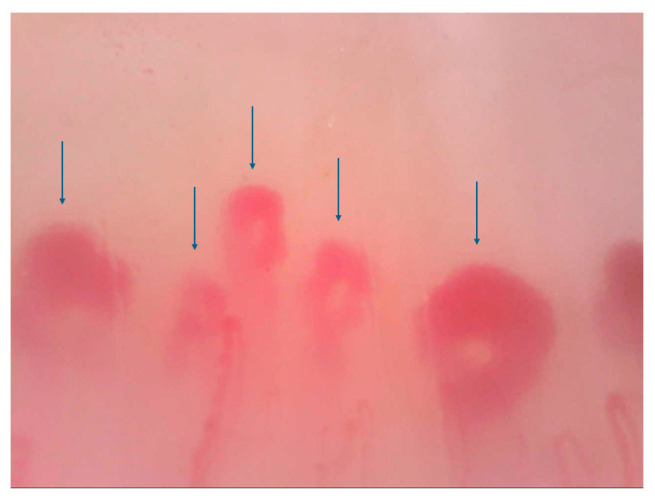
Nailfold videocapillaroscopy 200× (dimensions 1280 × 960). Scleroderma pattern example of total capillary count/mm at the last row. Arrows: megacapillaries.

All patients underwent NVC within 3 months before/after the HRCT scan, using a videocapillaroscope with a 200× magnification optical contact probe (DinoLite Digital Microscope, Dino-Lite Europe, Almere, the Netherlands). All fingers of both hands, excluding thumbs, were examined. At least four contiguous fields of 1 mm in the middle of the nailfold were captured from each finger [26]. The corresponding images were stored and analysed using Dino Capture 2.0 Software (version 1.5 14.b, AnMo Electronic Corporation, New Taipei City, Taiwan ). An experienced investigator (FF), blinded for the clinical data, reviewed and rated all NVC images. For each image, the total number of capillaries/mm was counted, regardless of morphology, as described for the “scleroderma pattern” [27]: U-shaped loops, tortuous capillaries, crossed loops, ramified/bushy capillaries, bizarre loops, giant capillaries (width of limbs > 50 μm), both homogenously and inhomogeneously shaped. Micro-haemorrhages and micro-thrombosis were excluded from the counting process. A consensus concerning image acquisition and analysis, scoring system and reliability of image acquisition and interpretation had already been reached by the authors (RDA, EC, FF) [26,28] (Figure 1). 

### 2.6. Statistical Analysis

All data were entered into a Microsoft Excel database and analysed with MedCalc^®^ version 18.6 (MedCalc Software, Ostend, Belgium). The values were expressed both as mean ± standard deviation (SD) or as median (interquartile range [IQR]). A two-sample “t” test was used to compare continuous variables and the χ^2^ test to compare categorical variables. The relationships among the lung analysis, the readers and the PFTs results were calculated using univariate regression analysis and Pearson’s product-moment correlation (Pearson r values). Furthermore, multivariate regression analyses were performed to identify factors associated with a higher percentage of pulmonary fibrosis at HRCT.

We included in the multivariate models the following covariates: age, sex, disease duration, anti-topoisomerase I antibodies, mRSS, m-Borg, GERD-Q, HAQ-DI, FVC, DLco (predicted) and capillary density (number of capillary/mm). Ethnicity was not included because all the enrolled patients were Caucasians.

The results were expressed as multivariate regression coefficient (R) and square regression coefficient corrected (R2) for the number of variables entered in the analysis. This enables the calculation and the predictivity of each multivariate model according to the number of variables entered into the model itself. The significance level was set at *p* < 0.05. The predictive performance of each variable was estimated by the area under the receiver operating characteristic curve (AUC-ROC). Youden’s index on the ROC curve analysis was used to determine the optimal cut-off point for the single variables.

A model based on Bayes’ theorem was then constructed to determine post-test probability using the pre-test probability of illness and the product of the positive likelihood ratio (+LR) of the items. The Fagan nomogram was used to graphically represent the model. The predictive variables included in the model are those that demonstrated significance in logistic regression analysis.

## 3. Results

The baseline characteristics (continuous variables) of the 78 SSc patients are summarised in Table 1.

The patients’ age ranged from 22 to 76 years.

Thirty-three (42.31%) patients showed HRCT features of ILD (Warrick score > 7, average total HRCT score = 11.62 ± 7.79). The mean disease extent score was 5.64 (±3.69), and the mean disease severity score was 5.97 (±4.03). Analysis of the ILD subtypes showed unclassifiable ILD in most patients (32.1%), with a combination of NSIP and UIP. No patient showed a diffuse alveolar damage (DAD) pattern, nor an organising pneumonia pattern [3,19,20].

Thirty-eight (48.7%) patients were classified as having dcSSc and forty (51.3%) patients as having lcSSc. Comparison of the two groups showed that patients with dcSSc were older (65.98 ± 10.30 vs. 60.97 ± 11.04 years; *p* < 0.05) and with a longer disease duration (12.04 ± 7.15 vs. 9.99 ± 8.56 years; *p* < 0.05) than those with lcSSc. ACA were present in 35 patients (44.8%) and anti-Scl-70 in 33 (42.3%). The proportion of anti-Scl-70 was significantly higher (*p* = 0.03) in patients with dcSSc.

FVC and DLco did not differ between patients with lcSSc and dcSSc. The time interval between PFTs and HRCT was 4.9 ± 1.6 days (range: 0–6 days).

On average, the HAQ-DI suggested a moderate functional impairment (0.96 ± 0.45). As expected, patients with dcSSc showed higher mean HAQ-DI scores than those with lcSSc (1.12 ± 0.39 vs. 0.81 ± 0.32; *p* < 0.05). The mean m-Borg Scale score was 2.73 (±2.22) and the Gerd-Q was 8.19 ± 4.03. Finally, the mean capillary number/mm was found to be 5.44 ± 2.22.

### Variables Associated with SSc-ILD

Table 2 reports the result of the multivariate regression analysis. DLco, M-Borg, GERD-Q and capillary density were significantly associated with the presence of ILD-SSc at HRCT. This model had a coefficient of determination R^2^ of 0.697. Age, gender, disease duration, anti-topo I antibodies, FVC predicted, HAQ-DI and mRSS were not significantly associated with ILD.

**Figure 2:**
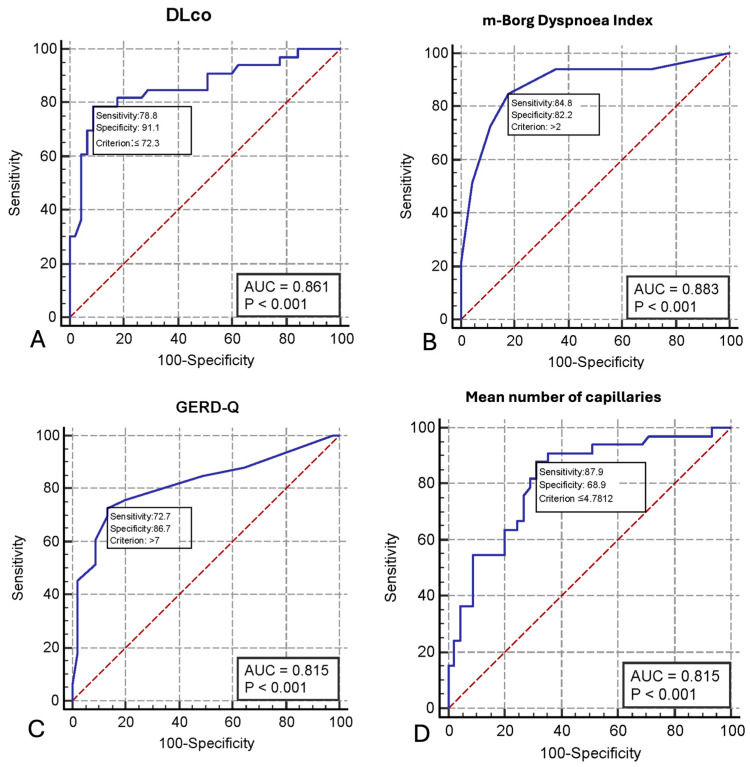
Receiver operating characteristic curve analysis showing the prognostic value of DLco (**A**), modified Borg Dyspnoea Scale (**B**), GERD-Q (**C**) and capillary density (n. of capillary/mm^2^) (**D**) on the discriminative performance for SSC-ILD disease.

The predictive performance of each variable was estimated by the AUC-ROC analysis (Figure 2A–D).

The DLco predicted had an AUC of 0.861 (95% CI 0.771–0.950; *p* < 0.001) and an optimal cut-off point of ≤72.3 (sensitivity 78.8%, specificity 91,1%, +LR 8.86) (Figure 2A) (Table A1, Appendix A). The m-Borg Scale demonstrated excellent discriminative ability, with an AUC of 0.883 (95% CI 0.801–0.966, *p* < 0.001) (Figure 2B) and an optimal cut-off point >2 (sensitivity 84.8%, specificity 82.2%, +LR 4.77) (Table A2, Appendix A). GERD-Q showed an AUC of 0.815 (95% CI 0.712–0.919, *p* < 0.001) and an optimal cut-off point >7 (sensitivity 72.7%, specificity 86.7%, +LR 5.45) (Figure 2C) (Table A3, Appendix B) and the capillary density showed an AUC of 0.815 (95% CI 0.718–0.911, *p* < 0.001) and an optimal cut-off point of ≤4.78 (sensitivity 87.9%, specificity 68.9%, +LR 2.82 (Figure 2D) (Table A4, Appendix B).

**Figure 3:**
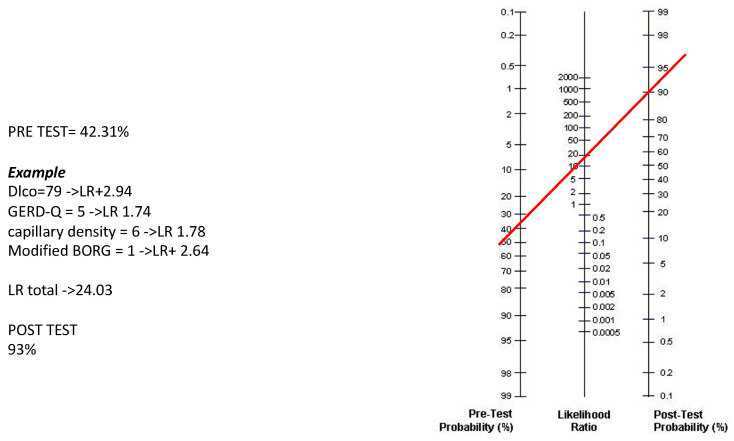
Example of application of the nomogram for calculation of the post-test probability. In the nomogram, the left axis represents the pre-test probability (42.31% in this case study), the middle axis represents the positive likelihood ratio and the right axis shows the post-test probability. To calculate the risk (post-test probability, %) of SSc-ILD in each patient, the positive likelihood ratio of each item in that patient has to be multiplied. The resulting positive likelihood ratio product represents the point intercepted on the middle axis (red line).

The four variables (DLco, m-Borg, GERD-Q and the mean capillary density/n. of capillary/mm) have been applied to the Fagan nomogram to calculate the post-test probability of having SSc-ILD. Calculation of the post-test probability is based on the pre-test probability (42.3% in our case series, which may vary depending on different local settings) and the product of the +LRs of the four items in the individual patient. Figure 3 shows an example of this calculation.

## 4. Discussion

Our study identified four variables indicating a significant association with SSc-ILD, namely DLco, m-Borg, GERD-Q and capillary density. Moreover, the concomitant inclusion of the single measures obtained in a post-test algorithm, tailored to these specific patient characteristics, significantly increases the likelihood of this association. 

All patients with SSc should be screened for ILD at the time of diagnosis [3,9], as SSc-ILD can be progressive, even in “lower risk” patients [7]. The estimated prevalence of ILD in SSc patients is highly variable depending on the population studied and the methodology used [3]. In a Canadian registry, 64% of 289 patients with SSc were diagnosed with ILD by HRCT, compared to 26% by physical examination and 22% by chest X-ray [29]. In a Norwegian population-based cohort of 650 patients with SSc, ILD at baseline was manifest on HRCT in half of the patients [30]. Our ILD prevalence was slightly lower (42.31%), presumably due to the single-centre design of the study and the type of methodology used for reading out the HRCT images, carried out by a radiologist and a rheumatologist with extensive experience in ILD evaluation [19,20,21].

Experts in SSc agree that comparative analysis of PFTs at the time of diagnosis may be relevant to estimate and predict SSc-ILD outcome [3,6,9,29,31], but PFTs alone are not sufficient as a tool for detecting patients with no obvious ILD on HRCT [3,31,32,33] since their reproducibility may vary [11,31,33] and they can be supplemented with additional information [30,31,32,33]. We found that a reduced DLco of 72.3% exhibits the best probability of being associated with SSc-ILD. Of note, DLco could be reduced if concomitant PAH and/or emphysema are present, but it should be mentioned that in our study, patients with a high probability of PAH according to the DETECT algorithm and/or PAH diagnosed by RHC and/or with chronic lung disease other than SSc-ILD were excluded [11,16,28,31,34].

Questionnaires assessing dyspnoea in SSc-ILD as well as the quality of life are used in clinical practice [10,20,35], especially for correlation with the extent of pulmonary involvement [10,20]. In our study, we found a significant ability of m-Borg in identifying patients with SSc-ILD, similar to the DLco. Obviously, m-Borg and DLCO are linked, one being the clinical counterpart of dyspnoea and the other reflecting alveolar-capillary exchanges, which are altered in ILDs [36,37]. 

Some studies suggested that gastro-oesophageal reflux may play a significant role in the pathogenesis of SSc-ILD [38,39,40]. Abnormalities in oesophageal peristalsis and the reduced oesophageal sphincter pressure are believed to lead to recurring micro-aspiration of gastric acid into the respiratory tract, resulting in chronic inflammation and progressive tissue damage [39,40,41]. Results of our study provide evidence that the self-administration of Gerd-Q, a validated questionnaire addressing the symptoms of gastro-oesophageal reflux [24,25,42], can be used to assess the presence of SSc-ILD, as this association is more likely when the score is >7.

Microvascular damage is one of the characteristic findings in SSc, which may occur early in the disease course. Moreover, it is thought to be strongly involved in the SSc-ILD pathogenesis [2,14,41,43]. A causal relationship between microcirculation abnormalities and pulmonary fibrosis could be found in the association of SSc-ILD with structural capillary changes detected by NVC [13,14]. Among the typical abnormalities belonging to the “scleroderma pattern”, capillary loss appears to be the one most correlated with ILD, through a qualitative (“active and “late” pattern) and quantitative assessment [13,44,45,46]. In particular, a significantly lower capillary density was reported in 48 patients with SSc-ILD diagnosed by HRCT [44] and, similarly, in 58 patients [45]. Our findings confirmed this association and also strengthened its value in identifying SSc-ILD when the number of capillaries is lower than 5/mm. This result is greatly useful, since capillary density was found to be the parameter with the highest inter-reader reliability [13,26,27] among other nailfold abnormalities, understanding that our NVC examination was obtained, as in both previous studies, using a videocapillaroscope with a 200× magnification contact lens [44,45].

It seems clear that the four measurements represent the clinical/biometric interface of the pathogenesis of pulmonary involvement, especially of the early phase of SSc (alteration of alveolar gas exchanges, chronic inflammation due to gastric micro-aspirations, chief role of autoimmune-type microcirculation damage as a primer of mesenchymal cell activation) [3,11,41,43] and appear suitable to be used as variables indicating SSc-ILD [11]. 

The lack of significance regarding age, gender, disease duration, mRSS and the presence of anti-topo I was similar to our previous findings [20]. The non-significant association with sex could be explained by the small number of male patients included in the study (67 F/11 M). Similarly, the lack of association with disease duration may be explained by recall bias. Further, mRSS results are not surprising, since this score has reproducibility issues [11,20]. Finally, the limitation of using a single centre with cross-sectional data could lead to selection bias. Moreover, our findings need to be validated in an external cohort. 

It should be emphasised that results obtained after the inclusion of the four-item measurements in a post-test algorithm further increase the probability of identifying patients with SSc-ILD. The inclusion of the four variables (DLco, m-Borg, GERD-Q and capillary density) was possible by using Fagan’s nomogram, which allows a prompt assessment in individual SSc patients, using the pre-test probability previously obtained. We performed simulations by entering the LR+ for the four variables into the algorithm, obtaining a higher post-test probability the more the variables differed from the normal. This calculation may be suitable to be performed using a smartphone app, following the example that was already published for COVID-19 pulmonary involvement [47]. Such a tool incorporating a set of input parameters may be usefully employed to assess the risk of lung tissue damage progression in longitudinal studies, even before evidence on HRCT [11,33].

## 5. Conclusions

There is a need for tools able to identify early SSc-ILD [9,11]. A comprehensive clinical assessment, including PFTs, an evaluation of respiratory symptoms and oesophageal involvement, and NVC, may ensure the identification of ILD, especially in those patients without clear evidence of interstitial disease at HRCT, or without the classic risk factors, or even in situations where a CT scan cannot be performed quickly, or in those awaiting the planned annual CT scan [7,9,36]. These measures are readily available in daily clinical practice [48], and may be incorporated in high-performance algorithms [9,13,28,48], providing more accurate and personalised prognostic information in future longitudinal studies [9,11,13,49].

## Figures and Tables

**Table 1:** Baseline study cohort characteristics of systemic sclerosis (SSc) patients.

Variables	Mean	SD	Median	25–75 P
Age (years)	63.60	10.34	65.00	56.00 to 71.00
Disease duration (years)	10.53	7.38	8.00	5.00 to 16.00
Modified Rodnan skin score	10.70	7.97	9.00	4.00 to 16.00
HRCT extent of disease score	5.64	3.69	3.00	3.00 to 9.00
HRCT severity of disease score	5.97	4.03	4.50	3.00 to 8.00
HRCT total score	11.62	7.79	6.00	5.00 to 18.00
DLco (% predicted)	73.41	16.76	78.15	59.00 to 88.00
FVC (% predicted)	87.72	18.51	88.95	76.00 to 103.00
HAQ-DI score	0.96	0.45	0.92	0.62 to 1.12
m-Borg score	2.73	2.22	2.00	1.00 to 5.00
Gerd-Q	8.19	4.03	6.00	5.00 to 11.00
Capillary density	5.44	2.22	5.15	3.43 to 7.65

SD = standard deviation; P = percentiles; FVC = Forced Vital Capacity; DLco = Single-breath Carbon Monoxide Diffusing Capacity of the Lung; HAQ-DI = Health Assessment Questionnaire-Disability Index; m-Borg: modified Borg Dyspnoea Scale; HRCT = High-resolution Computed Tomography. Gerd-Q = gastro-oesophageal reflux disease questionnaire.

**Table 2:** Summary of the results of multiple regression analysis, with regression coefficients for the variables.

Independent Variables	Coefficient	Std. Error	t	*p*	r_partial_	r_semipartial_
(Constant)	13.0435					
Age	0.0981	0.0851	1.153	0.253	0.1416	0.07871
Sex	0.1117	1.8317	0.061	0.951	0.0075	0.00416
Disease duration	−0.1681	0.1285	−1.309	0.195	−0.1602	0.08935
Anti-topoisomerase I	−0.0878	1.6181	−0.054	−0.956	0.0067	0.00370
modified Rodnan skin score	−0.02611	0.0990	−0.264	0.792	−0.0326	0.01800
DLco (% predicted)	−0.1101	0.0541	−2.035	0.045	−0.2448	0.13895
FVC (% predicted)	−0.0341	0.0386	−0.885	0.379	−0.1091	0.06041
HAQ-DI score	2.6693	1.5923	1.676	0.098	0.2036	0.11442
m-Borg	1.3327	0.5202	2.562	0.012	0.3029	0.17491
GERD-Q	0.6810	0.2819	2.416	0.018	0.2870	0.16497
Capillary density	−0.8847	0.4132	−2.141	0.036	−0.2567	0.14622

DLco = Single-breath Carbon Monoxide Diffusing Capacity of the Lung; Gerd-Q = gastro-oesophageal reflux disease questionnaire; HAQ-DI = Health Assessment Questionnaire-Disability Index; m-Borg: modified Borg Dyspnoea Scale; FVC = Forced Vital Capacity.

## Data Availability

Data are available from the corresponding authors on request.

## Appendix A

**Table A1:** Summary statistics of diffusing capacity for carbon monoxide (Dlco) performance.

ROC Curve								
Variable				DLco				
Classification variable				CUT-OFF ILD				
Sample size				78				
Positive group ^a^				33 (42.31%)				
Negative group ^b^				45 (57.69%)				
^a^ CUT-OFF ILD = 1								
^b^ CUT-OFF no ILD = 0								
Area under the ROC curve (AUC)								
Area under the ROC curve (AUC)				0.861				
Standard error ^a^				0.0457				
95% Confidence interval ^b^				0.771 to 0.950				
z statistic				7.887				
Significance level *p* (Area = 0.5)				<0.0001				
^a^ Hanley & McNeil. 1982								
^b^ AUC ± 1.96 SE								
Youden index								
Youden index J				0.6990				
Associated criterion				≤72.3				
Sensitivity				78.79				
Specificity				91.11				
Criterion values and coordinates of the ROC curve								
Criterion	Sensitivity	95% CI	Specificity	95% CI	+LR	95% CI	−LR	95% CI
≤55.2	30.30	15.6–48.7	97.78	88.2–99.9	13.64	1.8–101.4	0.71	0.6–0.9
≤56	36.36	20.4–54.9	95.56	84.9–99.5	8.18	2.0–34.1	0.67	0.5–0.9
≤60	60.61	42.1–77.1	95.56	84.9–99.5	13.64	3.4–54.3	0.41	0.3–0.6
≤61	60.61	42.1–77.1	93.33	81.7–98.6	9.09	2.9–28.1	0.42	0.3–0.6
≤66	69.70	51.3–84.4	93.33	81.7–98.6	10.45	3.4–31.9	0.32	0.2–0.5
≤67	69.70	51.3–84.4	91.11	78.8–97.5	7.84	3.0–20.5	0.33	0.2–0.6
≤72.3	78.79	61.1–91.0	91.11	78.8–97.5	8.86	3.4–23.0	0.23	0.1–0.5
≤74	78.79	61.1–91.0	82.22	67.9–92.0	4.43	2.3–8.5	0.26	0.1–0.5
≤74.3	81.82	64.5–93.0	82.22	67.9–92.0	4.60	2.4–8.8	0.22	0.1–0.5
≤78.5	81.82	64.5–93.0	73.33	58.1–85.4	3.07	1.8–5.1	0.25	0.1–0.5
≤79	84.85	68.1–94.9	71.11	55.7–83.6	2.94	1.8–4.7	0.21	0.09–0.5
≤82.2	84.85	68.1–94.9	48.89	33.7–64.2	1.66	1.2–2.3	0.31	0.1–0.7
≤84.5	90.91	75.7–98.1	48.89	33.7–64.2	1.78	1.3–2.4	0.19	0.06–0.6
≤85.4	90.91	75.7–98.1	40.00	25.7–55.7	1.52	1.2–2.0	0.23	0.07–0.7
≤88	93.94	79.8–99.3	37.78	23.8–53.5	1.51	1.2–1.9	0.16	0.04–0.6
≤90.2	93.94	79.8–99.3	22.22	11.2–37.1	1.21	1.0–1.4	0.27	0.06–1.2
≤91	96.97	84.2–99.9	22.22	11.2–37.1	1.25	1.1–1.5	0.14	0.02–1.0
≤92.2	96.97	84.2–99.9	15.56	6.5–29.5	1.15	1.0–1.3	0.19	0.03–1.5
≤93	100.00	89.4–100.0	15.56	6.5–29.5	1.18	1.0–1.3	0.00	0.04–1.6

**Table A2:** Summary statistics of the modified Borg Dyspnea Scale performance.

ROC Curve								
Variable				Modified Borg Dyspnea Scale				
Classification variable				CUT-OFF ILD				
Sample size				78				
Positive group ^a^				33 (42.31%)				
Negative group ^b^				45 (57.69%)				
^a^ CUT-OFF ILD = 1								
^b^ CUT-OFF no ILD = 0								
Area under the ROC curve (AUC)								
Area under the ROC curve (AUC)				0.883				
Standard error ^a^				0.0421				
95% Confidence interval ^b^				0.801 to 0.966				
z statistic				9.098				
Significance level *p* (Area = 0.5)				<0.0001				
^a^ Hanley & McNeil. 1982								
^b^ AUC ± 1.96 SE								
Youden index								
Youden index J				0.6707				
Associated criterion				>2				
Sensitivity				84.85				
Specificity				82.22				
Criterion values and coordinates of the ROC curve								
Criterion	Sensitivity	95% CI	Specificity	95% CI	+LR	95% CI	−LR	95% CI
>0.5	93.94	79.8–99.3	28.89	16.4–44.3	1.32	1.1–1.6	0.21	0.05–0.9
>1	93.94	79.8–99.3	64.44	48.8–78.1	2.64	1.8–4.0	0.094	0.02–0.4
>2	84.85	68.1–94.9	82.22	67.9–92.0	4.77	2.5–9.1	0.18	0.08–0.4
>3	72.73	54.5–86.7	88.89	75.9–96.3	6.55	2.8–15.4	0.31	0.2–0.5
>4	51.52	33.5–69.2	95.56	84.9–99.5	11.59	2.9–46.8	0.51	0.4–0.7
>5	21.21	9.0–38.9	100.00	92.1–100.0	12.29	3.1–48.9	0.52	0.7–0.9

## Appendix B

**Table A3:** Summary statistics of gastroesophageal reflux disease (GERD) questionnaire performance.

ROC Curve								
Variable				GERD-Q				
Classification variable				CUT-OFF ILD				
Sample size				78				
Positive group ^a^				33 (42.31%)				
Negative group ^b^				45 (57.69%)				
^a^ CUT-OFF ILD = 1								
^b^ CUT-OFF no ILD = 0								
Area under the ROC curve (AUC)								
Area under the ROC curve (AUC)				0.815				
Standard error ^a^				0.0528				
95% Confidence interval ^b^				0.712 to 0.919				
z statistic				5.964				
Significance level *p* (Area = 0.5)				<0.0001				
^a^ Hanley & McNeil. 1982								
^b^ AUC ± 1.96 SE								
Youden index								
Youden index J				0.5939				
Associated criterion				>7				
Sensitivity				72.73				
Specificity				86.67				
Criterion values and coordinates of the ROC curve								
Criterion	Sensitivity	95% CI	Specificity	95% CI	+LR	95% CI	−LR	95% CI
>4	87.88	71.8–96.6	35.56	21.9–51.2	1.36	1.1–1.8	0.34	0.1–0.9
>5	84.85	68.1–94.9	51.11	35.8–66.3	1.74	1.2–2.4	0.30	0.1–0.7
>6	75.76	57.7–88.9	80.00	65.4–90.4	3.79	2.0–7.0	0.30	0.2–0.6
>7	72.73	54.5–86.7	86.67	73.2–94.9	5.45	2.5–11.8	0.31	0.2–0.6
>8	69.70	51.3–84.4	86.67	73.2–94.9	5.23	2.4–11.4	0.35	0.2–0.6
>9	60.61	42.1–77.1	91.11	78.8–97.5	6.82	2.6–18.1	0.43	0.3–0.7
>10	51.52	33.5–69.2	91.11	78.8–97.5	5.80	2.1–15.6	0.53	0.4–0.8
>11	45.45	28.1–63.6	97.78	88.2–99.9	20.45	2.8–147.2	0.56	0.4–0.8
>14	18.18	7.0–35.5	97.78	88.2–99.9	8.18	1.0–64.8	0.84	0.7–1.0

**Table A4:** Summary statistics of mean capillary density performance.

ROC Curve								
Variable				Mean capillary density (number of capillary/mm^2^)				
Classification variable				CUT-OFF ILD				
Sample size				78				
Positive group ^a^				33 (42.31%)				
Negative group ^b^				45 (57.69%)				
^a^ CUT-OFF ILD = 1								
^b^ CUT-OFF no ILD = 0								
Area under the ROC curve (AUC)								
Area under the ROC curve (AUC)				0.815				
Standard error ^a^				0.0492				
95% Confidence interval ^b^				0.718 to 0.911				
z statistic				6.396				
Significance level *p* (Area = 0.5)				<0.0001				
^a^ Hanley & McNeil. 1982								
^b^ AUC ± 1.96 SE								
Youden index								
Youden index J				0.5677				
Associated criterion				≤4.78125				
Sensitivity				87.88				
Specificity				68.89				
Criterion values and coordinates of the ROC curve								
Criterion	Sensitivity	95% CI	Specificity	95% CI	+LR	95% CI	−LR	95% CI
≤2.53125	15.15	5.1–31.9	97.78	88.2–99.9	6.82	0.8–55.7	0.87	0.7–1.0
≤2.875	24.24	11.1–42.3	97.78	88.2–99.9	10.91	1.4–83.0	0.77	0.6–0.9
≤3	36.36	20.4–54.9	95.56	84.9–99.5	8.18	2.0–34.1	0.67	0.5–0.9
≤3.375	54.55	36.4–71.9	91.11	78.8–97.5	6.14	2.3–16.4	0.50	0.3–0.7
≤3.84375	54.55	36.4–71.9	80.00	65.4–90.4	2.73	1.4–5.3	0.57	0.4–0.8
≤4.09375	63.64	45.1–79.6	80.00	65.4–90.4	3.18	1.7–6.0	0.45	0.3–0.7
≤4.34375	66.67	48.2–82.0	73.33	58.1–85.4	2.50	1.5–4.3	0.45	0.3–0.8
≤4.53125	78.79	61.1–91.0	71.11	55.7–83.6	2.73	1.7–4.5	0.30	0.2–0.6
≤4.59375	81.82	64.5–93.0	68.89	53.4–81.8	2.63	1.7–4.2	0.26	0.1–0.6
≤4.78125	87.88	71.8–96.6	68.89	53.4–81.8	2.82	1.8–4.4	0.18	0.07–0.5
≤4.875	87.88	71.8–96.6	64.44	48.8–78.1	2.47	1.6–3.7	0.19	0.07–0.5
≤5.125	90.91	75.7–98.1	64.44	48.8–78.1	2.56	1.7–3.8	0.14	0.05–0.4
≤6.0625	90.91	75.7–98.1	48.89	33.7–64.2	1.78	1.3–2.4	0.19	0.06–0.6
≤6.09375	93.94	79.8–99.3	48.89	33.7–64.2	1.84	1.4–2.5	0.12	0.03–0.5
≤7.875	93.94	79.8–99.3	31.11	18.2–46.6	1.36	1.1–1.7	0.19	0.05–0.8
≤8.09375	96.97	84.2–99.9	28.89	16.4–44.3	1.36	1.1–1.7	0.10	0.01–0.8
≤8.59375	96.97	84.2–99.9	6.67	1.4–18.3	1.04	0.9–1.1	0.45	0.05–4.2
≤8.65625	100.00	89.4–100.0	6.67	1.4–18.3	1.07	1.0–1.2	0.00	0.06–4.5
